# Efficient CRISPR/Cas9-mediated gene disruption in the tetraploid protist *Giardia intestinalis*

**DOI:** 10.1098/rsob.210361

**Published:** 2022-04-27

**Authors:** Vendula Horáčková, Luboš Voleman, Kari D. Hagen, Markéta Petrů, Martina Vinopalová, Filip Weisz, Natalia Janowicz, Lenka Marková, Alžběta Motyčková, Vladimíra Najdrová, Pavla Tůmová, Scott C. Dawson, Pavel Doležal

**Affiliations:** ^1^ Department of Parasitology, Faculty of Science, Charles University, BIOCEV, Praha, Czech Republic; ^2^ Department of Microbiology and Molecular Genetics, College of Biological Sciences, UC Davis, Davis, CA, USA; ^3^ Institute of Immunology and Microbiology, First Faculty of Medicine, Charles University, Prague, Czech Republic

**Keywords:** *Giardia*, CRISPR/Cas9, gene knockout, multiploid

## Abstract

CRISPR/Cas9-mediated genome editing has become an extremely powerful technique used to modify gene expression in many organisms, including parasitic protists. *Giardia intestinalis*, a protist parasite that infects approximately 280 million people around the world each year, has been eluding the use of CRISPR/Cas9 to generate knockout cell lines due to its tetraploid genome. In this work, we show the ability of the *in vitro* assembled CRISPR/Cas9 components to successfully edit the genome of *G. intestinalis*. The cell line that stably expresses Cas9 in both nuclei of *G. intestinalis* showed effective recombination of the cassette containing the transcription units for the gRNA and the resistance marker. This highly efficient process led to the removal of all gene copies at once for three independent experimental genes, *mem*, *cwp1* and *mlf1.* The method was also applicable to incomplete disruption of the essential gene, as evidenced by significantly reduced expression of *tom40.* Finally, testing the efficiency of Cas9-induced recombination revealed that homologous arms as short as 150 bp can be sufficient to establish a complete knockout cell line in *G. intestinalis*.

## Graphical abstract

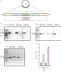

## 1. Introduction

*Giardia intestinalis* is a binucleated protist parasite that colonizes the small intestine of various mammals [1]. In humans, the parasite causes approximately 280 million cases of acute watery diarrhoea, giardiasis, annually [2]. *Giardia intestinalis* carries a tetraploid genome [3] with two sets of genes in both nuclei. The genome, however, can be modified by aneuploidy [4,5] and large regions of duplications [6] or deletions [7], which affect the copy number of a particular gene. The organism was long assumed to be asexual, but population genetics strongly suggest that recombination occurs between cells [8–11] through an unknown process. The low allelic sequence heterozygosity identified in *G. intestinalis* [12] may also be attributed to the exchange of DNA between the two-cell nuclei during a process recognized as diplomixis [13]. This unusual and largely unknown biology makes *G. intestinalis* a difficult target for reverse genetics.

Despite that, a palette of genetic tools has been developed for this parasitic protist, starting with the introduction of gene transfection techniques using plasmid or virus-mediated expression [14–17]. Morpholino oligonucleotides have been extensively used for translational repression of various genes, e.g. [18–22]. Recently, the catalytically inactive form of the CRISPR/Cas9 system, CRISPRi, has been introduced to *G. intestinalis* for the successful knockdown of several genes that encode cytoskeletal proteins [23] and recently, RNA-targeting Cas ribonuclease, CasRx, was experimentally used in *G. intestinalis* [24]***.*** The ‘classical’ CRISPR/Cas9 approach was used to reduce the gene expression in *G. intestinalis* [25,26] but this strategy did not achieve full genomic knockout. Thus, the only reported complete removal of all four gene copies was achieved for the *cwp1* gene using the Cre/loxP system [27]. However, this experimental application is laborious and time-consuming, as it relies on four cycles of consecutive gene deletions and plasmid curing [27].

The application of CRISPR/Cas9-based approaches is limited by DNA repair pathways that function in the target experimental organism [28]. It is mainly homologous recombination (HR) or the non-homologous end joining pathway (NHEJ) that define the experimental outcome of the CRISPR/Cas9 genome editing. According to genomic analysis, *G. intestinalis* lacks the components of NHEJ [29] and naturally or experimentally introduced double-strand breaks (DSB) must be repaired by HR.

Encouraged by the fact that the principles of CRISPR/Cas9-based systems are applicable to *G. intestinalis* [23,25], here, we attempted to develop CRISPR/Cas9 system for straightforward gene removal. Using an *in vitro* assembled Cas9-guideRNA ribonucleoprotein complex, we first confirmed that Cas9-mediated DSBs increase the efficiency of HR in the genome of *G. intestinalis.* After establishing a cell line with stable Cas9 expression, imported equally into both nuclei, we tested the ability of this cell line to delete target genes using an HR cassette that contains a resistance and gRNA expression cassette.

In fact, a single transfection of the gene-specific HR cassette was sufficient to mediate the removal of all copies of the gene from the *G. intestinalis* genome, as documented in three non-essential genes. The approach also proved to be applicable for knocking down the expression of an essential gene. Finally, the results showed that Cas9-induced DSB allows shorter homologous arms to be used for successful DNA recombination. Taken together, the combination of a cell line that stably expresses Cas9 in both nuclei and a single gene-specific recombination cassette offers a promising tool for straightforward functional genomics in *G. intestinalis*.

## 2. Results

### 2.1. Introducing the CRISPR/Cas9 system to *Giardia intestinalis*

**Figure 1.**
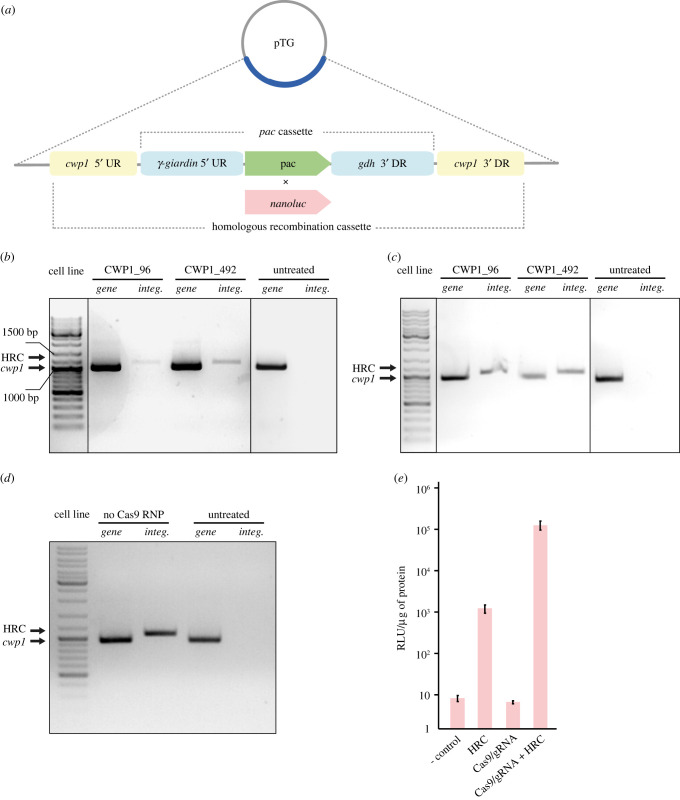
Recombinant Cas9-gRNA RNP increases the frequency of HR in *G. intestinalis.* (*a*) Schematic diagram of the pTG plasmid carrying the HR cassette. (*b*–*d*) Integration of the HR cassette into the *G. intestinalis* genome. Arrows indicate the PCR products used to detect the integration of the HR cassette (HRC) into the genome and the presence of the *cwp1* gene. Untreated wild-type cells (WBc6) were used as a control in all three experiments (*b*). Cells were electroporated with 50 µg of the pTG plasmid together with Cas9-gRNA RNP containing CWP1_96 or CWP1_492 gRNA. Cell lines were tested for HR cassette integration and the presence of *cwp1* in the genome. (*c*) Cells were electroporated with 3 µg of linear HR cassette together with Cas9-gRNA RNP complex and tested as above. (*d*) Cells were electroporated with 3 µg of linear HR cassette only and tested accordingly. (*e*) Effect of CRISPR/Cas9 on HR efficiency in *G. intestinalis.* Cells were electroporated with 3 µg of HR cassette carrying *nanoluc* without (HRC) or with Cas9-gRNA RNP (Cas9/gRNA + HRC). Electroporations containing only RNP (Cas9/gRNA) or water (- control) were used as controls. RLU, relative light units. The error bars represent the standard deviation of three independent experiments.

Based on the previous successful removal of all four copies of the *cwp1* gene from the *G. intestinalis* genome by the Cre/loxP system [27], the same target gene was chosen for the initial experiments with the CRISPR/Cas9 system. CWP1 is the main protein component of the infectious cyst of *G. intestinalis* [30] that is not expressed and therefore entirely dispensable in the trophozoite (active) stage of the parasite under normal cultivation conditions [27]. To assess whether the CRISPR/Cas9 system is suitable for genome editing in *G. intestinalis* as recently suggested [25], a ribonucleoprotein (RNP) complex containing a recombinant Cas9 protein preloaded with gRNA targeting *cwp1* was prepared *in vitro* [31]. The RNP was electroporated into the cells along with a pTG plasmid carrying HR cassette containing a transcription unit for the selection marker puromycin-*N*-acetyltransferase (*pac* cassette) [32] flanked by 988 bp and 998 bp of 5′ and 3′ upstream (UR) or downstream (DR) region of *cwp1*, respectively (figure 1*a*). Two *cwp1*-specific gRNAs were used, CWP1_96 or CWP1_492, targeting Cas9 nuclease to 96 bp or 492 bp position from the start codon of *cwp1*, respectively. After electroporation and puromycin selection, cells were grown to confluency and genomic DNA (gDNA) was extracted and tested by PCR for the integration of the HR cassette into the *G. intestinalis* genome and for the possible removal of *cwp1* (figure 1*b*). For both gRNAs, the successful integration of the HR cassette was detected; however, undisrupted *cwp1* was also detected in the cell population (figure 1*b*).

In an analogous experiment, the linear HR cassette was used as a template for the recombination in place of the pTG plasmid. To this end, the HR cassette was amplified by PCR (figure 1*a*) and electroporated into the cells along with Cas9-gRNA RNP. Again, PCR detection revealed successful integration of the cassette and the remaining *cwp1* in the cell population (figure 1*c*). Therefore, we attempted to isolate a cell line with complete knockout of *cwp1* by subcloning of single cells, but all generated clonal populations showed the presence of the gene (data not showed).

To test whether Cas9-induced DSB is responsible for the efficient integration of the HR cassette into the *G. intestinalis* genome, cells were electroporated with the cassette but without the addition of Cas9-gRNA RNP. Also, in this case, the HR cassette was integrated into the *G. intestinalis* genome, as detected by PCR performed on gDNA (figure 1*d*). This finding necessitated the need to clearly determine HR efficiency in the presence or the absence of Cas9-gRNA RNP. To this aim, selectable *pac* marker was replaced by the luciferase reporter gene (*nanoluc*) in the HR cassette (figure 1*a*). The cells were electroporated with different combinations of the components and the activity of NanoLuc was measured 24 h after electroporation (figure 1*e*). Background enzyme activity comparable to negative control (water) was detected when Cas9-gRNA RNP was delivered to cells without HR cassette. On the contrary, high luciferase activity was detected under both conditions involving the introduction of the HR cassette, yet in the presence of Cas9-gRNA RNP the activity was found to be higher by two orders of magnitude at the 24 h time point (figure 1*e*). These data suggested that the DSBs introduced by CRISPR/Cas9 increase the efficiency of HR in *G. intestinalis*. Recombinant Cas9-gRNA RNP can thus be used for plasmid-free editing of the *G. intestinalis* genome. However, at least in our hands, the approach was not sufficient to produce a cell line with complete gene knockout. One likely reason for the failure is the ineffective nuclear targeting of commercial recombinant Cas9. Thus, future studies involving recombinant Cas9-gRNA RNPs would likely have to include customized Cas9 containing a native nuclear localization signal (NLS) [23].

### 2.2. Stable expression of Cas9 in two nuclei of *Giardia intestinalis*

**Figure 2.**
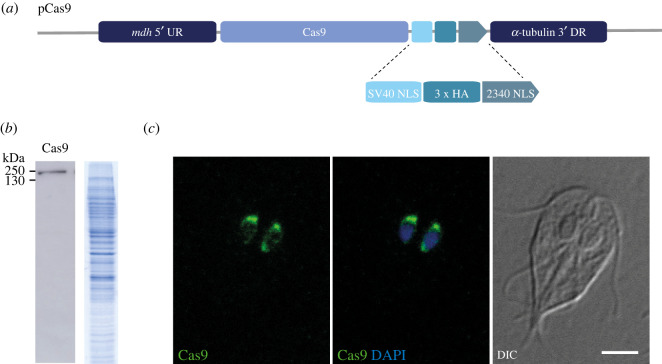
Expression of Cas9 in *G. intestinalis*. (*a*) Schematic diagram of the pCas9 plasmid. The chimeric Cas9 protein contains nuclear localization signals (NLS) from SV40, triple HA tag followed by NLS from GL50803_2340 at the C-terminus [23]. (*b*) The expression of Cas9 detected by anti-HA antibody in cell lysate (left), corresponding protein profile on Coomassie-stained SDS-PAGE gel (right). (*c*) HA-tagged Cas9 (green) is targeted to both nuclei stained with DAPI (blue), DIC, differential interference contrast. Scale bar corresponds to 5 µm.

Based on the results obtained from the use of recombinant Cas9, we attempted to increase the efficiency of HR by using a cell line endogenously expressing Cas9 in *G. intestinalis* [23]. The expression of chimeric Cas9 fused with the NLS of SV40, triple HA tag and the NLS of the *G. intestinalis* protein GL50803_2340 at its C-terminus was previously shown to mediate very specific nuclear localization of *Streptococcus pyogenes* Cas9 [23]. Therefore, the construct was subcloned into the pONDRA plasmid (figure 2*a*) [33]. Upon electroporation and G418 selection, the expression of Cas9 protein was confirmed by western blotting (figure 2*b*) and its nuclear localization was tested by immunofluorescence microscopy (figure 2*c*). The protein was readily detectable in the cell lysate fraction and microscopy revealed its localization in both *G. intestinalis* nuclei in agreement with the original report [23]. However, not all cells showed detectable expression of the protein. To increase the number of Cas9-positive cells and to obtain a homogeneous cell population for the following experiments, a clonal cell line was obtained from the transfected cell culture. Immunofluorescence analysis of the generated Cas9 cell line revealed that 67.8% (*N* = 403) of cells have detectable expression of the protein within both nuclei.

### 2.3. CRISPR/Cas9-mediated knockout of the *mem* gene present in only one of the two *Giardia intestinalis* nuclei

For the initial experiment, we took the advantage of the recently described absence of several genes from chromosome 5 in one of the nuclei of the used WBc6 cell line of *G. intestinalis* [7]. Hence, CRISPR/Cas9-mediated gene knockout was first tested by targeting the *mem* gene (GL50803_16374) estimated to be present in two or three copies within just one nucleus [7]. Moreover, *mem* encodes one of multiple high cysteine membrane proteins that are expressed on *G. intestinalis* surface [34] and therefore probably represents a non-essential gene.

**Figure 3.**
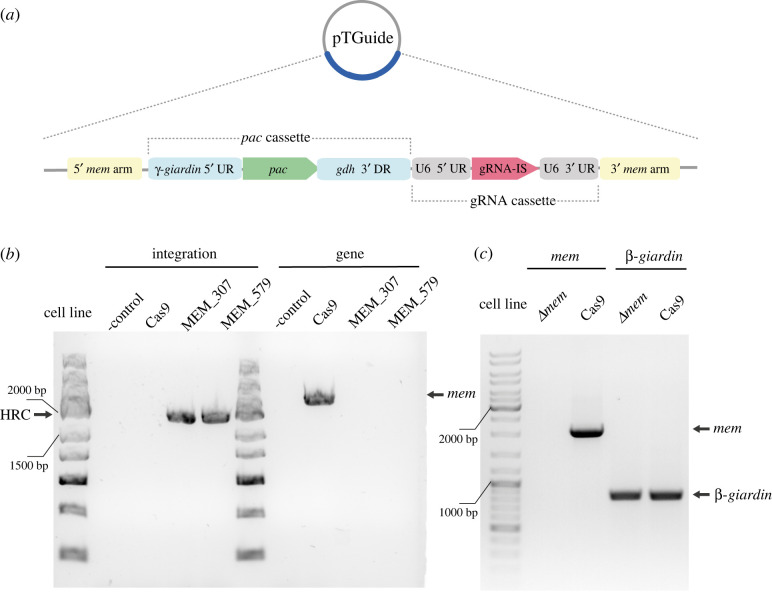
CRISPR/Cas9-mediated knockout of *mem* gene. (*a*) Schematic diagram of pTGuide plasmid carrying PAC cassette and guide RNA cassette including gRNA insertion site (gRNA-IS) used for all knockout experiments. (*b*) Cells carrying pCas9 were transformed with pTGuide containing one *mem* specific gRNA. Two gRNAs, MEM_307 and MEM _579, were tested. The transfectants were tested for HRC integration and the presence of *mem* in the genome (-control, water). (*c*) cDNA isolated form *Δmem* cells (*Δmem*)was tested for the presence of transcripts of *mem* gene and a control *β-giardin* gene. The parental Cas9-expressing cells (Cas9) were used as a control cell line.

This strategy allowed us to monitor multiple gene replacements by HR but occurring in only single nuclear compartment. To this end, the pTG plasmid was engineered to contain two transcription units. In addition to the *pac* cassette, a second unit for the transcription of gRNA containing 5′ UR and 3′ DR of U6 spliceosomal RNA was transferred from the dCas9g1pac plasmid [23]. These two cassettes were flanked by 1000 bp of the 5′ and 3′ homologous arms corresponding to 5′ UR and 3′ DR of the *mem* gene creating a pTGuide plasmid (figure 3*a*). Finally, two different gRNAs targeting the *mem* gene, MEM_307 and MEM_579, were inserted separately into the gRNA insertion site (gRNA-IS). The entire HR cassette was designed referring to the gene drive strategy of self-propagating CRISPR/Cas9 elements [35]. The starting assumption was that after the recombination of the HR cassette, the gRNA will no longer recognize this locus of the genome. Furthermore, continuous transcription of gRNA from the integrated cassette in Cas9-expressing cells would maintain the assembly of Cas9 RNP that would introduce DSB in the remaining alleles of the *mem* gene. In this case, the only available template for HR-mediated DSB repair would be the already recombined locus. This stepwise recombination would eventually lead to a complete gene knockout.

The pTGuide plasmid was electroporated into Cas9-expressing cells and when the transfectants reached confluency under puromycin and G418 selection, gDNA was isolated and tested for the HR cassette integration, as well as for the presence of the *mem* gene in the genome (figure 3*b*). Integration could be detected for both HR cassettes differing in the gRNA used. Interestingly, in both cases, no endogenous *mem* gene could be detected in the gDNA (figure 3*b*) indicating successful removal of all *mem* alleles from the genome. The intactness of the isolated gDNA was confirmed by PCR of a control *β-giardin* gene (figure 3*c*). The absence of the *mem* gene in the entire population of cells obtained after the antibiotic selection indicated high efficiency of HR that occurred in the transfected cells.

**Figure 4.**
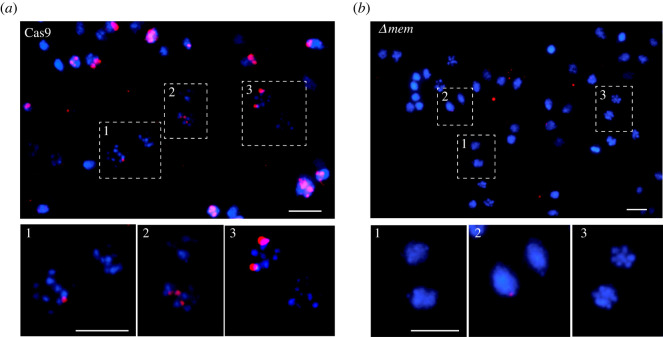
FISH analysis of *Δmem* cells. (*a*) Parental cells, in which Cas9 is expressed (Cas9), contain the *mem* gene only in one nucleus [7]. In the positive nuclei, usually (82%, *N* = 454) three distinct loci can be detected (inset 2), however, the number can vary in individual nuclei (e.g. insets 1 and 3). (*b*) In *Δmem* cells, the probe signal is barely visible (insets 1–3). Scale bars correspond to 10 µm.

Therefore, fluorescence *in situ* hybridization (FISH) was performed on parental and *Δmem* cells to check the completeness of gene knockout in the cell population. First, nuclear spreads of *G. intestinalis* expressing only Cas9 were used for hybridization with a 2000 bp long fragment of the *mem* coding sequence. Fluorescence detection confirmed the exclusive presence of the gene within one of the two nuclei (figure 4*a*). The observed hybridization frequency in the nuclei was 50.66% (*n* = 454 nuclei). Up to four, usually three (82%) signals were detected in the positive nuclei of the control cells (figure 4*a*). By contrast, in *Δmem* cells, the number of positive nuclei decreased to 7.41% (*n* = 364), and these showed only one signal per positive nucleus with poor hybridization intensity (figure 4*b*). Therefore, the average number of positive signals/loci per 100 cells dropped from 151.9 in control cells to eight in knockout cells, that is, 19 times. Internal hybridization control with probe against GL50803_17023 bound with the same intensity in control and knockout cells (electronic supplementary material figure S1).

Next, cDNA was prepared from *Δmem* cells and the presence of the corresponding mRNA was tested (figure 3*c*). While the transcript was present in the cDNA of the parental cell line, no signal was obtained using the cDNA isolated from *Δmem* cells. Finally, total protein samples of parental and *Δmem* cells were analysed by mass spectrometry to detect expression of the MEM protein. In accordance with the data obtained from the analyses of gDNA and cDNA, no peptide spectra were identified in *Δmem* cells, whereas the protein was detected in the control sample (electronic supplementary material, table S1). Taken together, these data showed that the CRISPR/Cas9 approach led to the complete removal of the *mem* gene from *G. intestinalis* genome. Interestingly, it could be proposed that due to the high efficiency of CRISPR/Cas9-triggered recombination in this case, the vast majority, if not all, of the cells lost all alleles of the targeted gene upon single electroporation and no further cell subcloning was required.

### 2.4. CRISPR/Cas9-mediated knockout of the *cwp1* and *mlf1* genes

Next, we tested the ability of the CRISPR/Cas9 system to eliminate the gene present in both *G. intestinalis* nuclei. Two genes were selected to test the robustness of the approach. In addition to the *cwp1* gene (see above) [27] a pTGuide plasmid was constructed to allow replacement of the *mlf1* gene. The latter was chosen because its CRISPR/Cas9-mediated knockdown in *G. intestinalis* was recently published [25] and, more importantly, a specific polyclonal antibody against Mlf1 was raised in our laboratory that would allow monitoring of Mlf1 protein levels after targeted genome editing. For both genes, two separate gRNAs (CWP1_96 and CWP1_492; Mlf1_163rc and Mlf1_491) were introduced into their HR cassettes to target Cas9 to the respective sequences in the genome.

Upon addition of the 5′ and 3′ homologous arms corresponding to 5′ UR and 3′ DR of the genes (988 bp for *cwp1* and 1000 bp for *mlf1* in the pTGuide plasmid), the plasmids were electroporated in Cas9-expressing cells. Based on the chance factor during the introduction of the recombination cassette into cells, two possible outcomes were expected. Puromycin-selected cells would carry the integrated cassette in either both nuclei or just one nucleus, since homologous chromosomes of the different nuclei are not known to recombine in the trophozoite stage. gDNA was isolated from cell lines derived from particular electroporations to test the integration of the HR cassette and the presence/absence of the genes by PCR.

**Figure 5.**
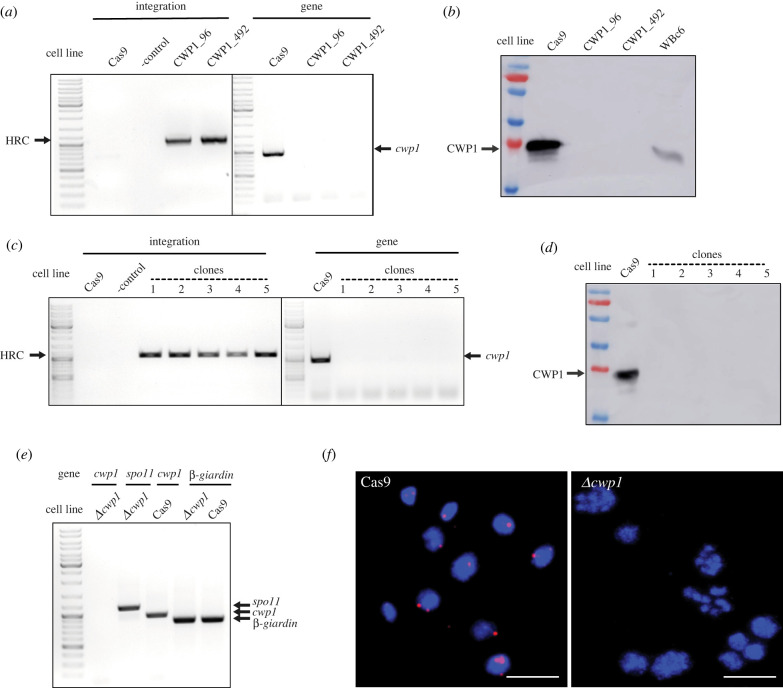
CRISPR/Cas9-mediated knockout of the *cwp1* gene. (*a*) Cas9-expressing cells were transformed with the pTGuide plasmid carrying CWP1_96 or CWP1_492 gRNA and tested for HR cassette integration (HRC) and presence of *cwp1*. Cas9-expressing cells (Cas9) or WBc6 cells were used as controls (water was used as the negative control for PCR) (*b*) The same cells were tested for CWP1 protein levels by western blot analysis using anti-CWP1 antibody. Cells were subjected to 22 h of encystation prior to analysis. (*c*) Five different clonal populations originating from cells carrying pTGuideCWP_492 were tested analogously by PCR for the integration of the HR cassette and the presence of the *cwp1* gene (water was used as the negative control (-control) for PCR) and (*d*) by western blot analysis for CWP1 protein levels. (*e*) *Δcwp1* cells were tested for the presence of *cwp1* cDNA. *spo11* and *β-giardin* were used as control genes for encystation-specific and constitutive transcription, respectively. (*f*) FISH analysis of *Δcwp1* cells. Representative images are shown. Nuclear spreads of parental Cas9-expressing cells (Cas9) were used as a positive control. The red signal corresponds to the hybridization of the *cwp1* probe. In the positive control, hybridization of the probe occurred in 91% of nuclei (*N* = 311), with one (31.4%), two (49.6%) or three signals (12.8%) per individual nuclei. In *Δcwp1* cells, only one weak signal was found in 2.4% of nuclei (*N* = 253), in the rest of the nuclei no or very weak non-specific staining was visible, showing the successful removal of *cwp1*. Scale bars correspond to 10 µm.

For *cwp1* CRISPR/Cas9, two cell lines derived from the two gRNAs used were selected for further analysis. Integration into the genome was detected in all three cell lines (figure 5*a*). Furthermore, the absence of the *cwp1* gene in all cell lines suggested that integration occurred in all four alleles of the gene. Therefore, the cells were *in vitro* induced for 22 h to undergo encystation and tested for the presence of the CWP1 protein. Western blot analysis of the cell lysate showed no detectable protein in any of the cell lines (figure 5*b*), although its expression could be observed in samples derived from parental cells carrying the plasmid pCas9 and the wild-type WBc6 strain of *G. intestinalis*.

To obtain a homogeneous cell population, five subclones originating from the CWP1_492 construct were expanded from single cells. Clonal populations were tested in the same way as the original cell line for the presence of the integrated cassette and the *cwp1* gene (figure 5*c*). The clones were also tested for the presence of CWP1 upon induction of the encystation (figure 5*d*). The results obtained from the five clones confirmed the complete elimination of the *cwp1* gene from the *G. intestinalis* genome. One of the clones, referred to as *Δcwp1*, was selected for further molecular analyses. The cDNA obtained from the encysting *Δcwp* cells was analysed for the presence of *cwp1, spo11* and β*-giardin* transcripts, of which the *spo11* gene served as an encystation-specific control [13]. Unlike the control sample, *cwp1* cDNA was not detected in *Δcwp1* cells, while it was identified for both *spo11* and *β-giardin* (figure 5*e*). FISH analysis performed on *Δcwp1* nuclei (figure 5*f*) revealed that 2.4% of the nuclei (*N* = 253) contained only one weak signal, while none or very weak non-specific staining was visible in the rest of the cells. However, comparative hybridization with the nuclei of the control cells occurred in 91% of the nuclei (*N* = 311), with one (31.4%), two (49.6%) or three signals (12.8%) per individual nucleus (figure 5*f*). Finally, in accordance with these data, no CWP1 peptides were detected in the proteomic analysis of encysting *Δcwp1* cells. The obvious conclusion of all these experiments was that the introduction of the engineered construct in pTGuide plasmid produced a complete knockout of *cwp1* in *G. intestinalis* stably expressing Cas9.

**Figure 6.**
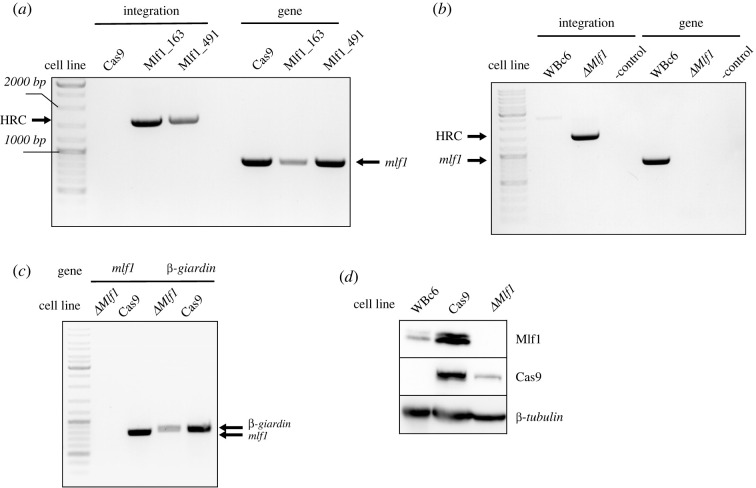
CRISPR/Cas9-mediated knockout of *mlf1*. (*a*) Cas9-expressing cells were transformed with the pTGuide plasmid carrying Mlf1 _163 or Mlf1_491 gRNA and tested for HR cassette integration (HRC) and the presence of *mlf1*. Two cell lines of each gRNA were tested. (*b*) The same cells were tested for Mlf1 protein level by western blotting using anti-Mlf1 antibody. Expression of Cas9 was also verified using anti-HA antibody. *β-tubulin* was used as a loading control. (*c*) *Δmlf1* clonal population derived from Mlf1_163 cells characterized as in (*a*). (*d*) *Δmlf1* cells were tested for the presence of *mlf1* cDNA by PCR. (*e*) The same cells were tested for Mlf1 protein level by western blotting using specific antibody. β-tubulin was used as a loading control*.* Cas9-expressing cells (Cas9) and wild-type WBc6 cells were used as controls (*a–e*).

In the case of *mlf1* CRISPR/Cas9, two cell lines derived from the two gRNAs used were tested. Upon gDNA isolation and PCR analysis, the integration of the HR cassette was detected in all resulting populations. The gene has not been completely removed from the genome, as we detected the presence of the *mlf1* gene in all cell lines (figure 6*a*)*.* Therefore, subclones were established from single cells and tested in the same way as mentioned above. Interestingly, one of the subclones obtained from Mlf1_163 gRNA was entirely devoid of the *mlf1* gene as tested by PCR on gDNA (figure 6*b*), nor was the *mlf1* transcript detectable in cDNA isolated from the clonal population (figure 6*c*). Furthermore, the Mlf1 protein was not detected in the cell lysate using a specific polyclonal antibody (figure 6*d*; electronic supplementary material, figure S2). Finally, the total protein content was analysed by mass spectrometry (electronic supplementary material, table S1) and in agreement with the immunoblotting results, no Mlf1-specific peptides were identified in the sample. Yet, the protein was identified in the parental Cas9-expressing cell line (electronic supplementary material, table S1). It could be concluded that the application of CRISPR/Cas9 produced a cell line with complete knockout of *mlf1* in *G. intestinalis.* Again, a single introduction of the recombination cassette into Cas9-expressing cells was efficient in removing all gene alleles.

### 2.5. CRISPR/Cas9-mediated knockdown of the *tom40* gene

Data obtained from CRISPR/Cas9-induced recombination at the *mlf1* locus indicated that integration of the HR cassette can result in a heterogeneous population of cells that differ by the number of replaced gene alleles. Furthermore, with regard to recently reported CRISPR/Cas9-mediated *mlf1* knockdown [25]*,* we tested whether a viable gene knockdown of an essential gene can be achieved through incomplete replacement of gene alleles. Tom40 is one of such essential proteins as it mediates the transport of proteins into the mitochondrial organelles of *G. intestinalis* known as mitosomes [36].

**Figure 7.**
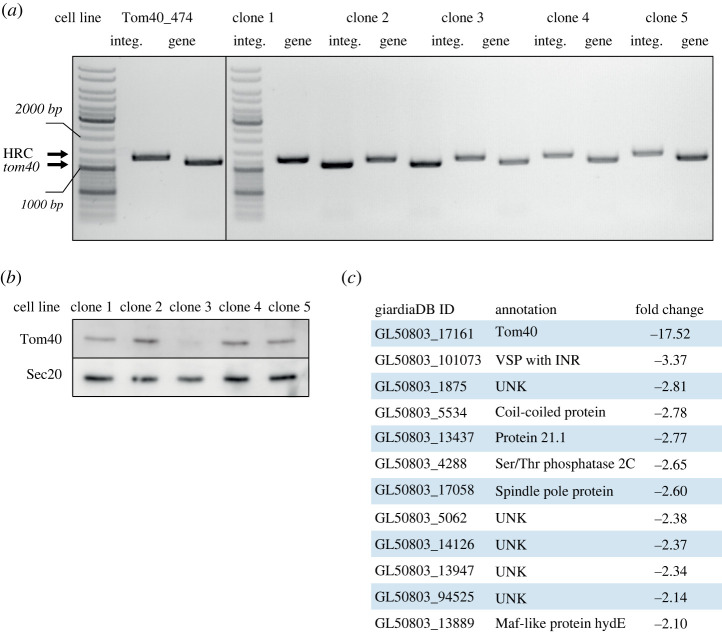
CRISPR/Cas9-mediated knockdown of *tom40*. (*a*) Cas9-expressing cells were transformed with the Tom40_474 gRNA-carrying pTGuide plasmid. The resulting population showed a successful integration of the cassette but also the remaining presence of *tom40* in its genome. Five subclones from the original population were further tested. (*b*) One clone (3) shows a decreased level of Tom40 protein. Sec20 protein was used as a loading control. The protein profiles of individual subclones are shown. (*c*) Top downregulated genes identified by comparative proteomic analysis of clone 3 and parental Cas9-expressing cell lines. (Full proteomic analysis is part of electronic supplementary material, table S1.)

Hence, the *tom40* gene was selected to be experimentally targeted by CRISPR/Cas9. To this end, *tom40*-specific gRNA (Tom40_474 gRNA) was inserted into the HR cassette that was flanked by 1000 bp of 5′ and 3′ UR and DR of the *tom40* gene in the pTGuide plasmid. The construct was then electroporated into Cas9-expressing cells. The resulting population was tested for HR cassette integration and the presence of *tom40* in the genome (figure 7*a*). Because all analysed cell lines still contained *tom40*, five subclones were established from the original population and analysed as described above. In all isolated cell lines, *tom40* was still present in the genome (figure 7*a*). Interestingly, though, the analysis of protein levels by immunoblotting revealed a significant decrease in the amount of Tom40 in one of the analysed subclones when compared to other subpopulations (figure 7*b*). Label-free proteomic quantification showed that the expression of the *tom40* gene was reduced to 5% of parental Cas9-expressing cells (electronic supplementary material, table S1). In this case, the CRISPR/Cas9 approach proved applicable to induce a knockdown of an essential gene. Therefore, the natural tetraploidy of *G. intestinalis* in combination with incomplete removal of gene alleles by CRISPR/Cas9 provides an experimental way to suppress gene expression by manipulation with gene dose. These data showed that CRISPR/Cas9 may actually represent an alternative strategy to CRISPRi [23] and CasRx [24] for gene silencing in *G. intestinalis*.

### 2.6. Testing the length of homologous arms needed for successful gene knockout

**Figure 8.**
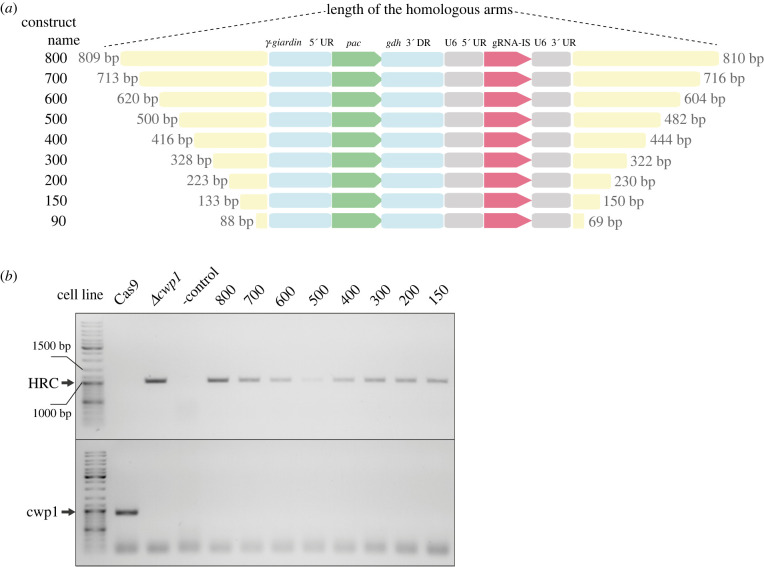
The length of homologous arms needed for successful recombination in the *G. intestinalis* genome. (*a*) Schematic diagram showing different lengths of homologous arms used in the HR cassette. (*b*) Cas9-expressing cells were transfected by pTGuide plasmid carrying CWP_492 gRNA and homologous arms of different lengths. The resulting strains were tested for the successful integration of the HR cassette and the presence of *cwp1*. Parental Cas9-expressing cells (Cas9) and *Δcwp1* cells were used as positive and negative controls for the presence of the gene, respectively.

The relatively high HR efficiency in the CRISPR/Cas9 experiments led us to define the shortest possible length of the homologous arms needed for the successful integration of the HR cassette into the genome *of G. intestinalis.* To this end, the *cwp1* gene was selected as a test locus. Ten different constructs (figure 8*a*) ranging from approximately 1000 bp to less than 100 bp of the homologous region were prepared and electroporated into Cas9-expressing cells. gDNA isolated from cell populations derived from particular electroporations was subjected to PCR analysis that detected HR cassette integration and removal of the *cwp1* gene (figure 8*b*). Integration was found in samples derived from all constructs down to the one with 133 bp and 150 bp of 5′ and 3′ homologous arms, respectively. Surprisingly, in all samples, full gene knockout was also detected. However, no integration or gene deletion was detected for the shortest construct with the 5′ and 3′ homologous arms of 88 bp and 69 bp, respectively (data not shown). These data indicated that when CRISPR/Cas9 is in place, the actual region for HR may be much shorter than used previously e.g. [15,27] and also in the initial experiments of this study.

## 3. Discussion

The discovery of the CRISPR/Cas9 system [37] soon after followed by its experimental application [38] revolutionized functional genomics of many organisms, including protist parasites [39]. It is the fundamental simplicity, transferability and mainly the precision of this two-component system that have enabled such explosive use and further methodological development [40]. *Giardia intestinalis*, as a tetraploid organism that also lacks the NHEJ pathway, has obviously been eluding the use of CRISPR/Cas9. However, two great advances have recently been made in the research of *G. intestinalis* biology. First, complete gene knockout based on HR combined with the recycling of the selection marker by the Cre/loxP system showed that it is possible, but far from easy, to establish gene deletion in *G. intestinalis* [27]. Second, the introduction of CRISPRi into *G. intestinalis* for transcriptional repression provided the first user-friendly editable molecular approach that takes advantage of the potential of the CRISPR/Cas9 system [23]. Importantly, the latter also defined highly specific and efficient endogenous NLS that targets Cas9 from *S. pyogenes* into both *G. intestinalis* nuclei [23]. Specifically, to the periphery of the nucleus, which resembles the presumed position of the nucleolus [41,42]. Importantly, the stable expression of Cas9 occurs without apparent toxicity to cells, which has caused problems in other systems, including protist parasites, e.g. [43,44].

Owing to the absence of the NHEJ pathway in *G. intestinalis,* DSB repair must involve HR. Likewise, in order to replace a gene in the *G. intestinalis* genome using CRISPR/Cas9, an alternate template must be provided to the molecular machinery that mediates HR. Therefore, in this work, a single HR cassette was designed to contain all the missing components needed for CRISPR/Cas9-mediated gene replacement. The construct included the transcription unit for puromycin-*N*-acetyltransferase as a selection marker and the transcription unit for a gene-specific gRNA [23]. Initially, homologous 1000 bp-long arms flanking these two units were used, but the introduction of a site-specific DSB using CRISPR/Cas9 appears to allow the use of fragments as short as 150 bp, as demonstrated for *cwp1.* The overall design of the HR cassette was inspired by self-propagating CRISPR/Cas9 elements used as gene drives in the experimental manipulations with population genetics [35]. The premise was that a successful integration can subsequently lead to the elimination of other gene alleles by CRISPR/Cas9-mediated DSB and HR-driven repair. Although some experiments using *cwp1* as a test gene indicate that such a process may happen in *G. intestinalis* nuclei, it is just as possible that all recombination events occur at once upon the electroporation of the construct.

However, no such results were obtained using transient *in vitro-*prepared Cas9 RNP. In this case, it was probably due to the ineffective nuclear targeting of the commercial Cas9, which lacked native NLS. Thus, for future experiments, it would be beneficial to use a customized enzyme containing the same NLS that was used for episomal expression.

In general, the observed efficiency of integration leading to gene knockout is highly encouraging for the establishment of further gene knockouts in *G. intestinalis*. Moreover, the CRISPR/Cas9 system was also permeable to allow the establishment of partial gene knockout that appeared as gene knockdown as recently published [25]. Undoubtedly, more studies are needed to understand the molecular background of the obtained results. Mainly, what is the actual sequence of molecular events that lead to complete gene knockout in *G. intestinalis*? And how do cells avoid the CRISPR/Cas9 system when incomplete gene knockouts are established in cells that still express both gRNA and Cas9? From an experimental point of view, it would be highly advantageous to implement the inducible expression of Cas9 to limit its possible and currently unknown non-specific activity in the *G. intestinalis* genome.

However, we believe that this work introduces the fully applicable CRISPR/Cas9 technique for routine use in the parasitic tetraploid protist *G. intestinalis.* It enables rapid establishment of complete gene knockout, which was not possible before, and offers the ability to perform functional genomic studies on this important parasite and exciting cell biology model.

## 4. Material and methods

### 4.1. Cell lines, cultivation, electroporation, subcloning and encystation

For all experiments, *G. intestinalis* WBc6 (ATCC 50803) cells were used. Cells were grown under anaerobic conditions in TYI-S-33 medium supplemented with 0.1% bovine bile (Sigma-Aldrich), 10% heat-inactivated adult bovine serum (Sigma-Aldrich) [45] and appropriate antibiotics at 37°C. Cells were electroporated using Bio-Rad Gene Pulser (BioRad) as described in [46]. The subcloning of *G. intestinalis* was carried out as follows: the fully grown *G. intestinalis* culture was placed on ice for 10 min to detach. Subsequently, 20 µl of cells were placed in 180 µl of TYI-S-33 medium in a 96-well tissue culture plate (VWR) and the same dilution step was performed five times. A drop of 2 µl from each dilution was checked for the number of *G. intestinalis* cells in three different wells to estimate the optimal dilution where only one to two cells per well were present. Twenty drops of 2 µl of cells from selected dilution were then placed on another 96-well plate in individual wells and the cell number was checked. The wells with only one cell were filled with growth medium and grown under anaerobic conditions at 37°C. After forming a monolayer, cells from individual wells were placed in cultivation tubes and grown normally. To ensure that the cloning was performed correctly, the whole procedure was repeated twice. Cells were subjected to encystation according to the Uppsala encystation protocol [47] in TYI-S-33 medium supplemented with 10% heat-inactivated adult bovine serum (Sigma-Aldrich) and 5 mg ml^−1^ of bovine bile (Sigma-Aldrich).

### 4.2. Guide RNA design and cloning

Guide RNAs (20 nt) were designed with the EuPaGDT design tool (http://grna.ctegd.uga.edu/) using the NGG PAM sequence. Off-target hits were checked against *G. intestinalis* Assemblage A genome [48]. gRNA oligonucleotides with four-base overhangs complementary to the plasmid sequence were annealed and cloned into BbsI digested pTGuide as described in [23]. The hybridization step was performed during the temperature gradient starting at 95°C for 5 min with a subsequent cooling of 1°C min^–1^ to 15°C.

### 4.3. Cloning and plasmid construction

The pTGuide plasmid was constructed as follows: the pTG plasmid [49] was modified by inserting the guide RNA expression cassette containing the guide RNA insertion site (gRNA-IS) surrounded by U6 snRNA 5′ UR and 3′ DR into the pTG plasmid. The cassette was amplified from dCas9g1pac plasmid [23] using gRNAcassette-F/R primers containing KpnI and ClaI restriction sites (electronic supplementary material, table S1). After cleavage with the respective enzymes, the cassette was ligated into the KpnI/ClaI-linearized pTG plasmid. Furthermore, the MluI/AvrII and ClaI/PacI sites were inserted at the 5′ and 3′ ends of a sequence encoding the puromycin acetyl transferase (PAC) cassette and the guide RNA expression cassette, respectively, for homologous arm cloning.

pCas9 was constructed as follows: *G. intestinalis* codon-optimized *S. p.* Cas9 nuclease followed by SV40 NLS, 3 x HA tag and NLS of GL50803_2340 together with 5′ UR of the *mdh* and 3′ DR of the *tubulin* genes were amplified from the pCas9U6g1pac plasmid [23] using primers Cas9-F/R. The PCR product was cleaved with KpnI and MluI restriction enzymes and cloned into a KpnI*/*MluI-linearized pONDRA plasmid [33].

pTGuide-CWP1 was constructed as follows: first, two guide RNAs (cwp1_96 and cwp1_492) were created by annealing the cwp1_96-F/R and cwp1_492-F/R primers, respectively, and inserted into separate pTGuide plasmids via BbsI restriction sites. Then, 988 bp of 5′ UR and 998 bp of 3′ DR of *cwp1* were amplified from *G. intestinalis* gDNA using cwp1_5_F/R and cwp1_3_F/R primers, respectively. The resulting products were cleaved by MluI/AvrII and ClaI*/*PacI restriction enzymes, respectively, and inserted into MluI*/*AvrII- or ClaI/PacI-linearized plasmid.

pTGuide-MEM was constructed as follows: first, two guide RNAs (mem_307 and mem_579) were created by annealing the mem_307_F/R and 579_F/R primers, respectively, and inserted into separate pTGuide plasmids via BbsI restriction sites. Then, 1000 bp of 5′ UR of *mem* was amplified from the *G. intestinalis* gDNA using mem_5_F and R primers. The resulting sequence was cleaved by MluI and AvrII restriction enzymes and inserted into the MluI*/*AvrII-linearized plasmid. The 3′ flanking region was created by joining two PCR products by overlap extension polymerase chain reaction (OE-PCR). First, the *pac* cassette together with the *mem* guide RNA cassette was amplified from pTGuide using the pac_cassette_F and gRNA_cassette_R primers. Then 1000 bp of 3′ DR of *mem* was amplified from *G. intestinalis* gDNA using the mem_3_F and _R primers. PCR products from reactions 1 and 2 were used as a template for the third PCR reaction with primers pac_cassette_F and mem_3_R. Based on the overhangs created in reactions 1 and 2, a complete 3′ flanking region was created and resulting sequence was cleaved with AvrII and PacI restriction enzymes and inserted into the AvrII/PacI-digested plasmid.

pTGuide-Mlf1 was constructed as follows: first, two guide RNAs (Mlf1_163rc and Mlf1_491) were created by annealing the mlf1_163rc_F/R and mlf1_491_F/R primers, respectively, and inserted into separate pTGuide plasmids via BbsI restriction sites. Then, 1000 bp of 5′ UR and 3′ DR of *mlf1* were amplified from *G. intestinalis* gDNA using mlf_5_F/R and mlf_3_F/R primers, respectively. The resulting products were cleaved by MluI/AvrII and ClaI*/*PacI restriction enzymes, respectively, and inserted into MluI*/*AvrII- or ClaI/PacI-linearized plasmid.

pTGuide-Tom40 was constructed as follows: at first, the tom40_474_F/R primers (electronic supplementary material, table S1) were annealed to create tom40_474 guide RNA that was inserted into pTGuide plasmid via BbsI restriction sites, forming pTGuide_474 plasmid. Then, 1000 bp of 5′ UR and 3′ DR of *tom40* (GL50803_17161) were amplified from the *G. intestinalis* gDNA using tom40_5_F/R and tom40_3_F/R primers, respectively (electronic supplementary material, table S1). The resulting products were cleaved with respective enzymes and subsequently cloned into pTGuide_474 plasmid via MluI/AvrII and ClaI/PacI restriction sites, respectively.

To test homologous arm lengths, primers HA_X_F/R (X stands for the number of base pairs) were used to prepare particular fragments of the 5′ and 3' regions (electronic supplementary material, table S1); 5′ homologous arm with HA_X_F and cwp1_5_R primers, while 3′ homologous arm with cwp1_3_F and HA_X_R primers.

The HR cassette containing *nanoluc* was amplified from pTG-NLuc (figure 1*a*) where *pac* was replaced by *nanoluc*. Owing to the presence of internal restriction sites the *nanoluc* gene was amplified by OE-PCR from two PCR fragments. The first fragment was amplified from pTG_CWP1 using Nluc_F_I and Nluc_R_I primers, and the second from the *G. intestinalis* codon-optimized *nanoluc* gene using Nluc_F_II and Nluc_R_II primers. Two PCR products were used as a template for the third PCR reaction with Nluc_F_I and Nluc_R_II primers. The resulting PCR product was cleaved by EcoRV/XhoI and ligated into the pTGuide plasmid.

### 4.4. cDNA preparation

mRNA from encysting and non-encysting KO strains was isolated by NucleoTrap mRNA Mini Column Kit (Macherey-Nagel) according to the manufacturer's protocol, with the additional step of passing the cell lysates through a 0.9 mm needle (20 gauge) six times. Then, 60 ng of purified mRNA was used for the preparation of cDNA using the SuperScript VILO cDNA Synthesis Kit (Invitrogen) in 20 µl reaction volume according to the manufacturer's protocol. For subsequent PCR, 1 µl of cDNA was used. PCR was performed using SaphireAmp Fast PCR Master Mix (Takara) in 50 µl volume.

### 4.5. Testing the HR cassette integration and the presence of a target gene

For testing the integration of individual HR cassettes by PCR, primer pairs contained the same reverse primer specific to pTGuide (pTGuide_int_R) and a gene-specific forward primer: cwp1_int_F, mem_int_F, mlf1_int_F, and tom40_int_F. To test the presence of individual genes in the *G. intestinalis* genome, the following primers were used: for *mem*, *cwp1* and *mlf1*, mem_genomic_F/R, CWP_genomic_F/R and mlf_genomic_F/R were used, respectively. For *tom40*, primers tom40_genomic_F/R were used (electronic supplementary material, table S1).

### 4.6. Genome editing by exogenous Cas9-RNP complexes

Alt-R® CRISPR-Cas9 crRNA (CRISPR RNA) (cwp1_96: GAAATCTACGATGCCACTGA, cwp1_492: ACAGCTGATTGCAGTCTAGG) and tracrRNA (trans-activating crRNA) were obtained from Integrated DNA Technologies and Alt-R® *S. pyogenes* Cas9 nuclease V3 was provided by the same company. Cas9-RNP complexes were prepared according to the company protocol (0.6 µl 100 µM crRNA, an equal amount of tracrRNA and 1.3 µl 62 µM Cas9 nuclease were used for each reaction). Cas9-RNP complexes were delivered to *G. intestinalis* using a standard electroporation protocol [46]. When only the HR cassette was used as a template for HR, it was amplified from the pTG plasmid using HRC_F and HRC_R primers (electronic supplementary material, table S1).

### 4.7. Luciferase assay

NLuc luminescence was monitored using the Nano-Glo® Luciferase Assay System (Promega) according to the manufacturer's protocol. Briefly, cells were collected 24 h after transfection and mixed with an equal volume of Nano-Glo® Luciferase Assay reagent and relative luminescence (RLU) was measured after 7 min using GloMax® 20/20 Luminometer (Promega). The identical cell lysate was then used for the determination of protein concentration using the Bicinchoninic Acid Protein Assay Kit (Sigma-Aldrich) according to the manufacturer's protocol. Sample absorbance measurements were carried out on a Shimadzu UV-1601 spectrophotometer using UV Probe 1.10 software.

### 4.8. Fluorescence *in situ* hybridization

The FISH protocol and probe construction were carried out according to Tůmová *et al*. [7]. Briefly, the chromosome spreads were prepared as described by Tůmová *et al*. [5]. The FISH hybridization mixture (20 ng of the labelled probe, 10 µg of salmon sperm, 50% deionized formamide (Sigma-Aldrich)) in 2x SSC was incubated on slides at 82°C for 5 min. Single colour FISH was developed by a TSA Plus TMR system according to the manufacturer's protocol (PerkinElmer) using a digoxigenin-labelled probe and an anti-dig HRP antibody (Roche Applied Science). In two-colour FISH, sequential double hybridization signal development was processed according to the manufacturer's protocol (PerkinElmer), as a combination of (i) a digoxigenin-labelled probe, anti-dig-HRP antibody and TSA-Plus TMR and (ii) a biotin-labelled probe, streptavidin-HRP and TSA-Plus Fluorescein. For the FISH probe design, primers mem_fish_F/R and cwp1_fish_F/R were used, respectively (electronic supplementary material, table S1). For internal hybridization control, a probe against GL50803_17023 was used in combination with *mem* probe and GL50803_17495 in combination with *cwp1* probe [5,7]. The PCR products of approximately 2000 bp inside the ORFs of the above listed genes (for *cwp1* the whole ORF of 726 bp was used) were cloned into a pJET 1.2/blunt cloning vector (Fermentas) and transformed into chemically competent TOP10 *E. coli* cells (Invitrogen). Purified PCR products amplified from plasmids isolated from a single bacterial colony (QIAprep Spin MiniprepKIT, Qiagen) were labelled by random priming with digoxigenin-11-dUTP (Roche) or biotin-11-dUTP (PerkinElmer) using a DecaLabel DNA Labelling Kit (Fermentas). Controls for FISH accuracy were conducted as described in [5,7].

### 4.9. Mass spectrometry

#### 4.9.1. Protein digestion

Cell pellets were homogenized and lysed at 95°C for 10 min in 100 mM TEAB (triethylammonium bicarbonate) containing 2% SDC (sodium deoxycholate), 40 mM chloroacetamide, 10 mM TCEP (Tris(2-carboxyethyl)phosphine) and further sonicated (Bandelin Sonoplus Mini 20, MS 1.5). Protein concentration was determined using BCA protein assay kit (Thermo) and 30 µg of protein per sample was used for MS sample preparation. Samples were further processed using SP3 beads according to Hughes *et al*. [50]. Briefly, 5 µl of SP3 beads was added to 30 µg of protein in lysis buffer and filled to 50 µl with 100 mM TEAB. Protein binding was induced by addition of ethanol to 60% (vol./vol.) final concentration. Samples were mixed and incubated for 5 min at RT. After binding, the tubes were placed into magnetic rack and the unbound supernatant was discarded. The beads were then washed two times with 180 µl of 80% ethanol. After washing, the samples were digested with trypsin (trypsin/protein ration 1/30) reconstituted in 100 mM TEAB at 37°C overnight. After digestion, samples were acidified with TFA to 1% final concentration and peptides were desalted using in-house made stage tips packed with C18 disks (Empore) according to Rappsilber *et al*. [51].

#### 4.9.2. nLC-MS2 analysis

Nano reversed phase columns (EASY-Spray column, 50 cm × 75 µm ID, PepMap C18, 2 µm particles, 100 Å pore size) were used for LC/MS analysis. Mobile phase buffer A was composed of water and 0.1% formic acid. Mobile phase B was composed of acetonitrile and 0.1% formic acid. Samples were loaded onto the trap column (C18 PepMap100, 5 µm particle size, 300 µm × 5 mm, Thermo Scientific) for 4 min at 18 µl min^−1^. The loading buffer was composed of water, 2% acetonitrile and 0.1% trifluoroacetic acid. The peptides were eluted with a Mobile phase B gradient of 4–35% B in 120 min. Eluted peptide cations were converted to gas-phase ions by electrospray ionization and analysed on a Thermo Orbitrap Fusion (Q-OT-qIT, Thermo Scientific). Survey scans of peptide precursors from 350 to 1400 *m/z* were performed in orbitrap at 120 K resolution (at 200 *m/z*) with a 5 × 10^5^ ion count target. Tandem MS was performed by isolation at 1.5 Th with the quadrupole, HCD fragmentation with normalized collision energy of 30, and rapid scan MS analysis in the ion trap. The MS2 ion count target was set to 10^4^ and the maximum injection time was 35 ms. Only those precursors with charge state 2–6 were sampled for MS2. The dynamic exclusion duration was set to 45 s with a tolerance of 10 ppm around the selected precursor and its isotopes. Monoisotopic precursor selection was activated. The instrument was run in top-speed mode with 2 s cycles [52].

#### 4.9.3. Data analysis

All data were analysed and quantified with MaxQuant software (v. 1.6.3.4) [53]. The false discovery rate was set to 1% for both proteins and peptides and a minimum peptide length was set to seven amino acids. The Andromeda search engine was used for the MS/MS spectra search against the *G. intestinalis* protein database (downloaded from https://giardiadb.org on 11 July 2020). Enzyme specificity was set as C-terminal to Arg and Lys, also allowing cleavage at proline bonds and a maximum of two missed cleavages. Dithiomethylation of cysteine was selected as fixed modification and N-terminal protein acetylation and methionine oxidation as variable modifications. The ‘match between runs’ feature of MaxQuant was used to transfer identifications to other LC-MS/MS runs based on their masses and retention time (maximum deviation 0.7 min) and this was also used in quantification experiments. Quantifications were performed with the label-free algorithm in MaxQuant [54]. Data analysis was performed using Perseus 1.6.1.3 software [55].

### 4.10. Immunofluorescent labelling and microscopy analysis

For immunofluorescence, the cells were labelled as described [56]. Briefly, the cells were fixed by 1% paraformaldehyde for 30 min at 37°C. The cells were then centrifuged at 900*g* for 5 min at RT and washed in PEM buffer (200 mM PIPES, 2 mM EGTA, 0.2 mM MgSO_4_, pH 6.9). Then, the cells were resuspended in PEM buffer and transferred to poly-l-lysine-coated coverslips and let to attach for 15 min. The cells were then permeabilized by 0.2% Triton X-100 in PEM buffer for 20 min and washed three times with PEM buffer. Then, the cells were blocked by PEMBALG (PEM supplemented with 1% BSA, 0.1% NaN_3_, 100 mM lysine and 0.5% gelatin) for 30 min. All blocking and immunolabelling steps were performed in a humid chamber at room temperature. After blocking, the cells were incubated in PEMBALG containing monoclonal anti-HA antibody produced in rat (1 : 1000 dilution, Roche) for 1 h. The coverslips were then washed three times for 15 min in 0.1% Tween-20 in PBS and incubated in PEMBALG containing monoclonal anti-rat antibody conjugated with Alexa488 (1 : 1000 dilution, Life Technologies) for 1 h. The coverslips were then washed three times for 15 min in 0.1% Tween-20 in PBS and mounted in Vectashield containing DAPI (Vector Laboratories).

Static images were acquired on Leica SP8 FLIM inverted confocal microscope equipped with 405 nm and white light (470–670 nm) lasers and FOV SP8 scanner using HC PL APO CS2 63x/1.4 NA oil-immersion objective. Laser wavelengths and intensities were controlled by a combination of AOTF and AOBS separately for each channel. Emitting fluorescence was captured by internal spectrally tunable HyD detectors. The imaging was controlled by the Leica LAS-X software. Maximum intensity projections and brightness/contrast corrections were performed in FIJI ImageJ software. For FISH experiments, Olympus BX51 fluorescence microscope equipped with a DP70-UCB camera was used; imaging sensitivity was set up equally for controls and knockout cell lines.

## Data Availability

All data are part of this submission, either in the main body or in the electronic supplementary material [57].
